# N-acetylcysteine as a novel methacrylate-based resin cement component: effect on cell apoptosis and genotoxicity in human gingival fibroblasts

**DOI:** 10.1186/s12903-024-03988-2

**Published:** 2024-02-12

**Authors:** Yang Yang, Liping Wang, Zelun Huang, Lingu Ge, Jianwei Shi

**Affiliations:** 1 Department of Oral Implantology, School and Hospital of Stomatology, Guangdong Engineering Research Center of Oral Restoration and Reconstruction & Guangzhou Key Laboratory of Basic and Applied Research of Oral Regenerative Medicine, Guangzhou Medical University, Guangzhou, China; 2 School and Hospital of Stomatology, Guangdong Engineering Research Center of Oral Restoration and Reconstruction & Guangzhou Key Laboratory of Basic and Applied Research of Oral Regenerative Medicine, Guangzhou Medical University, Guangzhou, China; 3 Suzhou Stomatological Hospital, Suzhan Lu 1366, Suzhou, 215000 China; 4 Department of Orthodontics, School and Hospital of Stomatology, Guangdong Engineering Research Center of Oral Restoration and Reconstruction & Guangzhou Key Laboratory of Basic and Applied Research of Oral Regenerative Medicine, Guangzhou Medical University, Guangzhou, China

**Keywords:** DNA double-strand breaks, Methacrylate-based resin cement, N-acetylcysteine

## Abstract

**Background:**

N-acetylcysteine (NAC) reduces the cytotoxicity and genotoxicity induced by monomers leached from dental composite resins. Herein, we investigated the effects of methacrylate-based resin cement used in dental implant restoration on apoptosis and genotoxicity, as well as the antiapoptotic and antigenotoxic capabilities of its component, NAC.

**Methods:**

The antioxidant NAC (0.1 or 1 wt.%) was experimentally incorporated into the methacrylate-based dental resin cement Premier®. The Premier® + NAC (0.1 or 1 wt.%) mixture was subsequently immersed into Dulbecco’s modified Eagle’s medium for 72 h, and used to treat human gingival fibroblasts (HGFs). The viability of HGFs was determined using the XTT assay. The formation of deoxyribonucleic acid (DNA) double-strand breaks (DNA-DSBs) was determined using a γ-H2AX assay. Reactive oxygen species (ROS), apoptosis, necrosis, and cell cycles were detected and analyzed using flow cytometry.

**Results:**

The eluate of Premier® significantly inhibited HGF proliferation in vitro by promoting a G1-phase cell cycle arrest, resulting in cell apoptosis. Significant ROS production and DNA-DSB induction were also found in HGFs exposed to the eluate. Incorporating NAC (1 wt.%) into Premier® was found to reduce cell cytotoxicity, the percentage of G1-phase cells, cell apoptosis, ROS production, and DNA-DSB induction.

**Conclusion:**

Incorporating NAC (1 wt.%) into methacrylate-based resin cement Premier® decreases the cell cytotoxicity, ROS production, and DNA-DSBs associated with resin use, and further offers protective effects against the early stages of cell apoptosis and G1-phase cell cycle arrest in HGFs. Overall, our in vitro results indicate that the addition of NAC into methacrylate-based resin cements may have clinically beneficial effects on the cytotoxicity and genotoxicity of these materials.

## Background

Implant restorations can be either screw- or cement-retained [1]. Cement-retained implant restorations are generally preferred by dentists because of their ease of fabrication, superior esthetics, and lower risk of mechanical complications than those due to screw-retained restorations [2]. However, these implants are associated with a risk of excess cement retention in the peri-implant soft tissue, resulting in peri-implant mucositis and peri-implantitis [3]. Further, a prior study presented moderate certainty evidence to indicate a similar risk for peri-implant mucositis and peri-implantitis between screw- and cement-retained restorations [4].

Compared with other dental luting materials, resin cements, which are permanent luting agents used in implant-based prostheses, are insoluble and have superior mechanical and physical qualities [5, 6]. Additionally, resin cements can be applied to most abutment-prosthesis combinations (e.g., zirconia abutments and crowns) [6].

Dental resin cements comprise a methacrylate-based resin matrix and inorganic fillers [5]. According to a survey of dental schools in the United States, methacrylate-based components are the most frequently used luting agents for cementing implant restorations [7]. However, the polymerization of methacrylate-based resins is incomplete and residual (co) monomers can be leached from the cements [8], resulting in direct contact with the gingiva. Mutagenicity, embryo toxicity, and teratogenicity caused by the released monomers have been reported [9]. Excess methacrylate-based cement is significantly associated with clinical inflammation and development of peri-implant diseases [10].

Our prior study demonstrated that the eluted components of composite resins can induce cytotoxicity and deoxyribonucleic acid (DNA) double-strand breaks (DNA-DSBs) in human gingival fibroblasts (HGFs )[11]. Moreover, the (co) monomers triethylene glycol dimethacrylate (TEGDMA) and 2-hydroxyethyl methacrylate (HEMA) induce reactive oxygen species (ROS) production and HGF apoptosis [12, 13] Considering that oxidative DNA damage in fibroblasts is closely related to periodontitis [14, 15], a recent study indicated that the pathogeneses of peri-implantitis and periodontitis are similar [16]; hence, ROS production, cell apoptosis, and oxidative DNA damage may play a vital role in peri-implantitis. As such, evaluating methacrylate-based resin cement-induced apoptosis and genotoxicity in HGFs may be highly relevant.

N-acetylcysteine (NAC) is a radical scavenger [17], which has been shown to reduce the cytotoxicity induced by TEGDMA and HEMA [13, 18]. Our previous studies demonstrated that the addition of NAC to cell culture media reduced the genotoxicity of dental (co) monomers and their epoxy metabolites [19–21]. Moreover, the incorporation of NAC into dental composite resins may supply sufficient amounts of released antioxidants [22].

NAC in prepolymerized polymethyl methacrylate exerts protective effects against cell apoptosis and genotoxicity [23]. However, few studies have focused on the incorporation of NAC in dental luting agents and their effects on the surrounding tissue. Therefore, to address this knowledge gap, we aimed to investigate the incorporation of NAC into methacrylate-based resin cement used in implant restoration.

## Methods

### Sample preparation

Samples were prepared in accordance with the method outlined in our previous study [22]. Briefly, 1 wt.% (20 mg), 0.1 wt.% (2 mg), and 0 wt.% (0 mg; control group) of grounded fine powder NAC (Sigma-Aldrich, China) was added to 2 g of uncured methacrylate-based resin cement (premier®, Plymouth Meeting, PA, USA). To achieve the most homogeneous distribution possible, NAC was experimentally introduced into the uncured resin cement using a dental spatula. The mixture was subsequently compressed into a polytetrafluoroethylene ring with a diameter of 10 mm and thickness of 2 mm, and mounted on a plastic matrix strip.

Samples were transferred to a 24-well plate with 1 mL of Dulbecco’s modified Eagle’s medium (DMEM) (Gibco, USA) containing 10% fetal calf serum (Gibco, USA) after a 4–5 min curing period at room temperature.

### Cell culture

HGFs were purchased from Provitro GmbH (Berlin, Germany). HGFs (passage 8) were cultured in 175 cm^2^ cell culture flasks (BD Falcon, USA) at 37 °C and 100% humidity with 5% CO_2_, in DMEM media supplemented with 10% fetal bovine serum (Gibco, USA) and 1% penicillin/streptomycin (Gibco, USA). After reaching confluency, the cells were washed with Dulbecco’s phosphate-buffered saline (Gibco, USA) and detached from the flasks by treatment with trypsin/EDTA (Gibco, USA).

### XTT-based viability assay

The viability of HGFs was assessed using an XTT assay according to the ISO 10993-5 standards. This assay was performed in accordance with earlier research [11, 20, 24]. In brief, a 96-well microtiter plate was seeded with HGFs at a density of 20,000 cells/well in 100 μL of medium. The cells were subsequently exposed to the eluate of Premier® or the eluate of Premier® + NAC (0.1 or 1 wt.%) before being incubated for 24 h at 37 °C with 5% CO_2_ and 100% humidity. The only treatment administered to the control cells was the medium; 1% Triton X-100 (Sigma-Aldrich) was used as a negative control. Optical density (OD) was measured using a microplate reader (Multiskan FC; Thermo Fisher Scientific, Shanghai, China) and spectrophotometry at 450 nm. Three separate experiments (*n* = 3) were performed in triplicate. The following equation was used to determine cell viability:

1 $$\textrm{Cell}\ \textrm{viability}\ \left(\%\right)=\frac{\textrm{OD}\ \textrm{of}\ \textrm{test}\ \textrm{group}}{\textrm{OD}\ \textrm{of}\ \textrm{control}\ \textrm{group}}\times 100$$

### Formation of ROS

A ROS Assay Kit (Beyotime, China) was used to measure ROS production following the manufacturer’s instructions. HGFs were extracted to create cell suspensions after undergoing various pretreatments (Premier® or Premier® + NAC (0.1 or 1 wt.%), 24 h at 37 °C). HGFs were then treated with 10 μM dichloro-dihydro-fluorescein diacetate for 30 min at 37 °C. A minimum of 10^4^ cells were analyzed for each sample. Fluorescence was measured at 485-nm excitation and 520-nm emission using a flow cytometer (Agilent Technologies, Santa Clara, CA, USA).

### γ -H2AX immunofluorescence

The γ-H2AX assay was used to evaluate the production of DNA-DSBs in HGFs. The methodology presented in our earlier studies was used in this assay [11, 20, 24]. In brief, 12-mm round cover slips were dispersed in a 24-well plate after being washed with 1 N HCl. In each well, HGFs were seeded at a density of 7 × 10^4^ cells/mL in the medium, followed by an overnight incubation at 37 °C. The eluate of Premier® or Premier® + NAC mixtures was applied to the cells for 6 h. Only the medium was added to the negative control cells. After 15 min, 1 mM H_2_O_2_ (Sigma-Aldrich) was added to the medium as a positive control. Immunofluorescence staining was performed as previously described [20]. Laser scanning confocal microscopy (Zeiss, Göttingen, Germany) was used to capture fluorescent images. The image acquisition and data analysis were based on the procedures described in our previous study [20].

### Detection of apoptosis and necrosis

Apoptosis and necrosis were assessed using an Annexin V-FITC Apoptosis Detection Kit (Invitrogen), following the manufacturer’s instructions. HGFs were harvested with PBS/EDTA and collected by centrifugation after being submitted to various treatments (Premier® or Premier® + NAC (0.1 or 1 wt.%), 24 h at 37 °C). After labeling with annexin V-FITC and propidium iodide (PI), apoptotic or necrotic cells were detected, and the fluorescence intensity was promptly examined using flow cytometry (Agilent, NovoCyte Quanteon, USA) at 488-nm excitation and 520-nm emission.

### Cell cycle analysis

An Invitrogen Cell Cycle and Apoptosis Analysis Kit was used to determine cell cycle progression. In brief, HGFs were resuspended in a solution containing RNase and PI after being subjected to various treatments (Premier® or Premier® + NAC (0.1 or 1 wt.%), 24 h at 37 °C), and fluorescence was measured using a flow cytometer (Agilent Technologies, Santa Clara, CA, USA).

### Statistical analysis

The mean and standard deviation for each value was calculated from three separate experiments performed in triplicate. All values were tested for normal distribution using the Shapiro–Wilk test. GraphPad Prism 5 software (San Diego, CA, USA) was used to assess group comparisons using one-way or two-way analysis of variance and Tukey’s post-hoc test for normally distributed data. The Kruskal–Wallis test and post-hoc analysis (Dunn’s test) were used when the data were not normally distributed. Differences were considered significant at *p* < 0.05.

## Results

### XTT assay

**Fig. 1:**
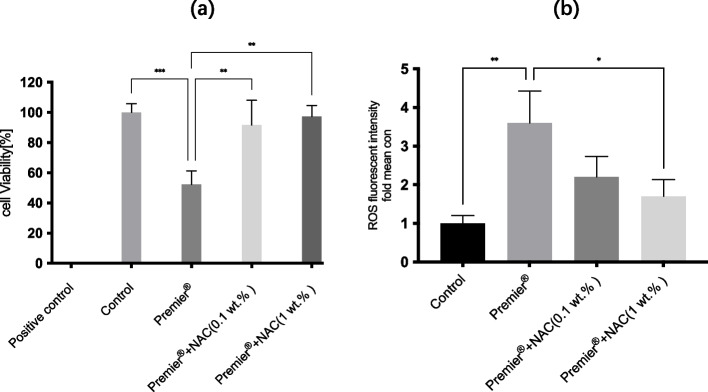
Cell viability and ROS production of HGFs treated with Premier® or Premier® + NAC. **a** HGFs viability in XTT assay after incubation with the eluates of Premier®, Premier® + NAC(0.1 wt.%)and Premier® + NAC(1 wt.%)for 24 h. Control cells received medium only, positive control cells were treated with 1% Triton X-100. Data are expressed as percentage of control (Eq. (1)) and represent mean ± SD (*n* = 3); ^∗∗^ *p* < 0.01; ^∗∗∗^ *p* < 0.001 (**b**) Intracellular ROS level evaluated by flow cytometry after a 24 h exposure period. Data represent mean ± SD (*n* = 3); ^∗^ *p* < 0.05; ^∗∗^ p < 0.01

HGFs exposed to the eluates of the investigated resin cement showed significantly lower cell viability than untreated HGFs (52.32 ± 8.98%, *p* < 0.001). The incorporation of NAC (0.1 wt.%, 1 wt.%) significantly protected the cell viability against Premier®-induced cell death compared to the cell death observed in controls (91.77 ± 16.28%, 97.31 ± 7.38% vs. 52.32 ± 8.98%, *p* < 0.01, Fig. 1a).

### Formation of ROS

A 3.6-fold increase in ROS levels was found for Premier® compared to that associated with the control, representing a significant increase (*p* < 0.01, Fig. 1b), Conversely, a 3.6-fold significant reduction of ROS accumulation was detected in the Premier® + NAC (1 wt.%) group compared to that associated with the Premier® group, representing a significant reduction (*p* < 0.05, Fig. 1b). However, no significant ROS reduction was found for Premier® + NAC (0.1 wt.%).

### γ -H2AX immunofluorescence

**Fig. 2:**
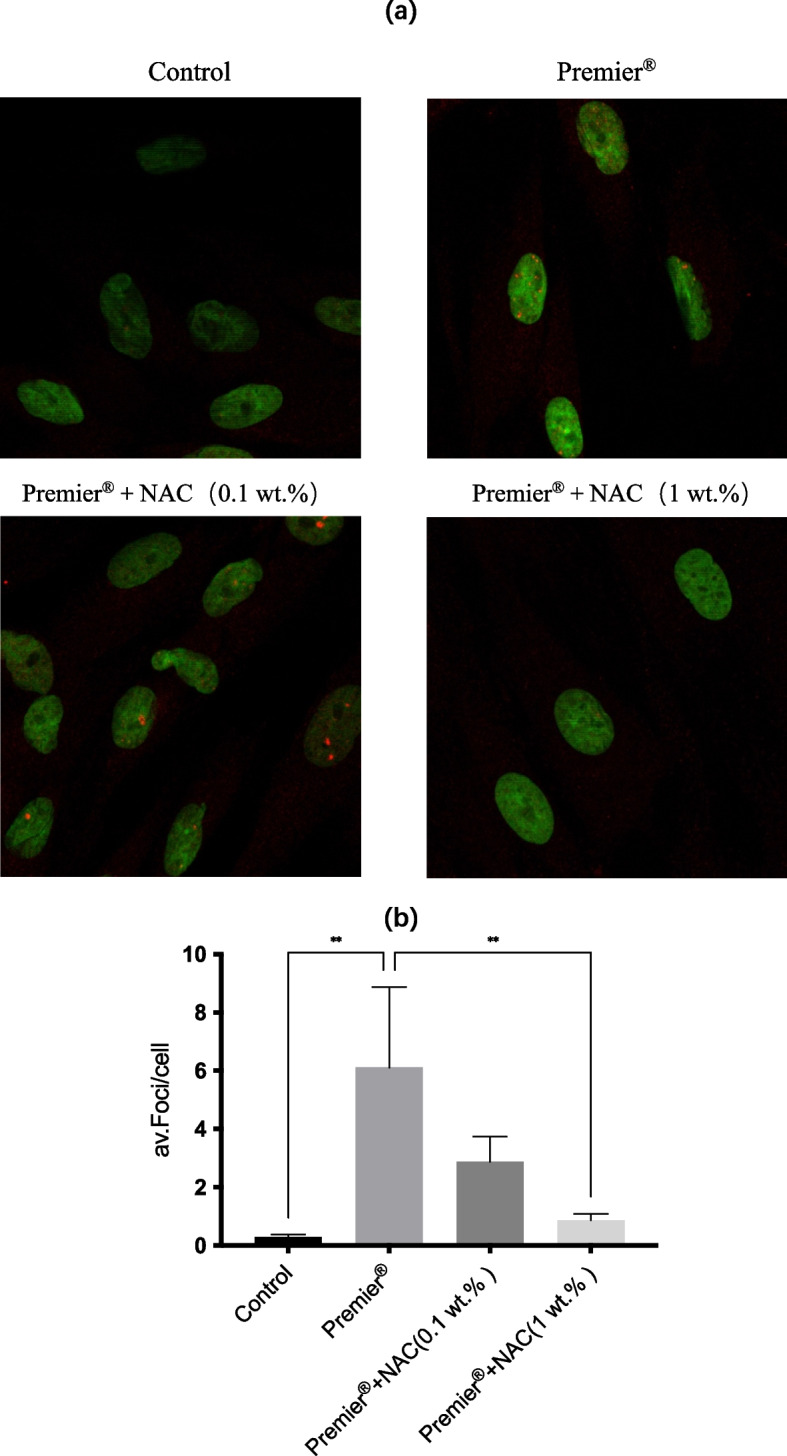
DNA-DSBs induced by Premier® and protective effects of incorporated NAC. **a** Representative images of immunofluorescent staining for H2AX phosphorylation (orange) in HGFs, after exposure to different substances compared to control cells. Sybr green (green) is a marker for DNA and stains the whole nucleus of the cell. Nucleus of HGFs with typical γ-H2AX-specific foci can be observed in Premier® and Premier® + NAC(0.1 wt.%)treated groups. **b** Average of induced γ-H2AX DSBs-foci/cell in HGFs treated with different samples. Data are expressed as mean ± SD (*n* = 3); ^∗∗^ *p* < 0.01

H_2_O_2_ (1 mM) induced 24.66 ± 3.66 DSB-foci/cell in positive controls. Medium induced 0.29 ± 0.08 DSB-foci/cell in the negative control. The eluate of Premier® induced a significantly higher number of DSB-foci (6.11 ± 2.76 foci/cell, *p* < 0.01) compared to those observed in the control. The greatest DSB-foci reduction was associated with Premier® + NAC (1 wt.%), which induced a 7-fold lower number of foci/cell compared to those observed in the Premier® group (0.87 ± 0.22 foci/cell, p < 0.01), no significant reduction in DSB-foci (2.88 ± 0.86) was associated with Premier® + NAC (0.1 wt.%). Representative images of immunofluorescent staining for **γ**-H2AX are shown in Fig. 2.

### Cell apoptosis assay

**Fig. 3:**
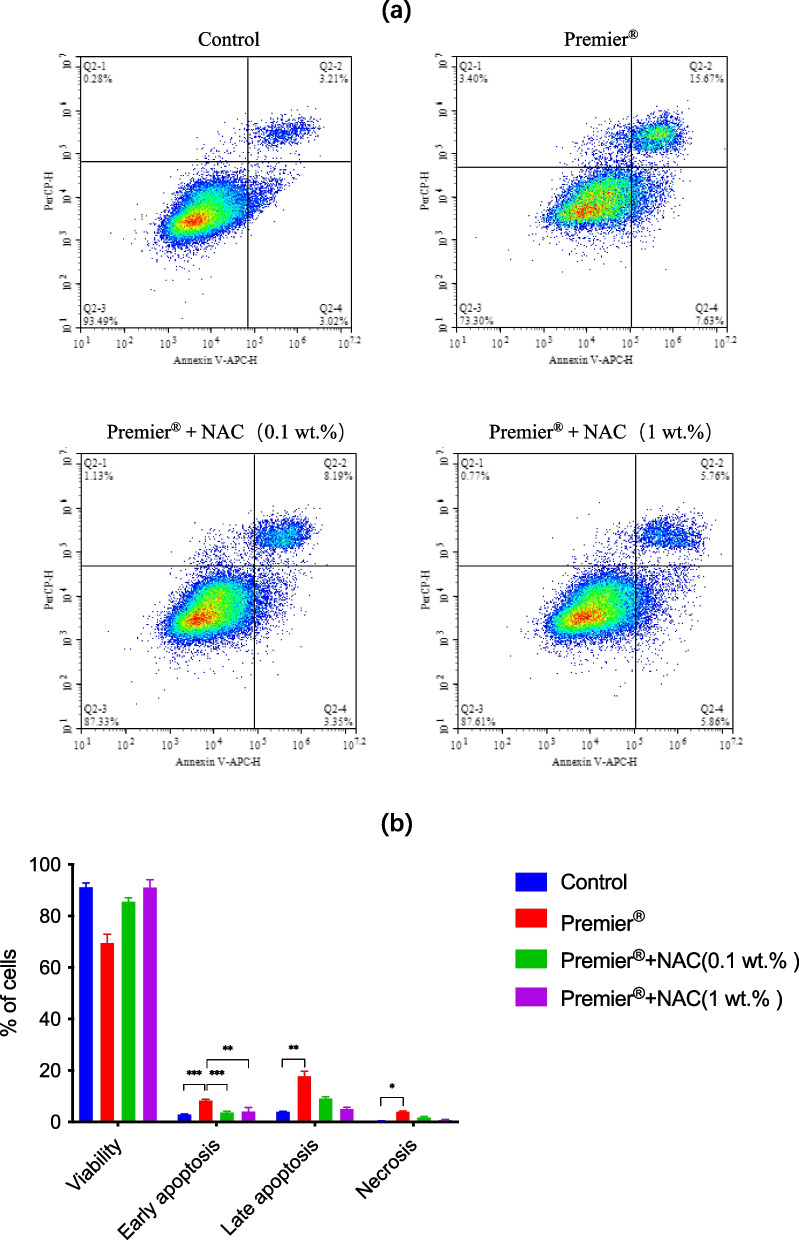
Induction of apoptosis and necrosis of HGFs treated with Premier® or Premier® + NAC. Cells were stained with annexin V- FITC/propidium iodide (PI) and analyzed by flow cytometry. **a** The percentages of viable cells (unstained), early apoptotic cells (Annexin), late apoptotic cells (Annexin + PI) and necrotic cells (PI) of one typical experiment are denoted in the quadrants of each density blot. **b** Bar graphs represent flow cytometry data. Data represent mean ± SD (n = 3); ^∗^ p < 0.05; ^∗∗^ p < 0.01; ^∗∗∗^ *p* < 0.001

As shown in Fig. 3, untreated cultured cells showed a viability of approximately 91%, with minimal signs of apoptotic cell death. Exposure of cultured cells to the eluate of Premier® reduced the percentage of viable cells to 69.47% and increased the number of cells in the various phases of cell death. A marked (*p* < 0.001) increase of early apoptotic cells was observed following treatment with the eluate of Premier® (8.25 ± 0.55%), compared to controls (2.84 ± 0.19%). The incorporation of NAC (0.1 wt.% or 1 wt.%) significantly (p < 0.001; *p* < 0.01) attenuated the number of cells in early apoptosis (3.58 ± 0.56%; 4.07 ± 1.55%, respectively, as compared to Premier® group). After 24 h of incubation with the eluate of Premier®, late apoptotic cell population drastically (p < 0.01) increased to 17.73 ± 1.20% as compared with control cells (3.85 ± 0.25%). Moreover, exposure of cultured cells to the eluate of Premier® resulted in a significant (*p* < 0.05) increase in cell necrosis (3.83 ± 0.38%), as compared to controls (0.34 ± 0.11%).

### Cell cycle analysis

**Fig. 4:**
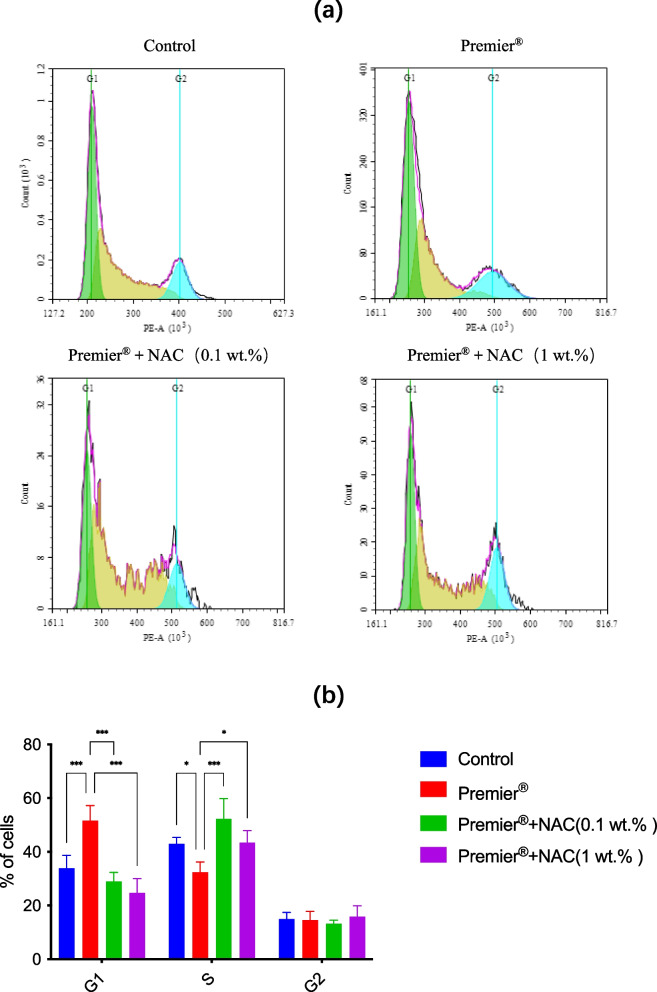
Cell cycle arrest induced by Premier® and protective effects of incorporated NAC. **a** Cell cycle analysis of HGFs exposed to control or Premier® + NAC(0.1 wt.%)and Premier® + NAC(1 wt.%)for 24 h. **b** Bar graphs represent flow cytometry data. Data represent mean ± SD (n = 3); ^∗^ *p* < 0.05; ^∗∗∗^ *p* < 0.001

As shown in Fig. 4, exposure of HGFs to the eluate of Premier® significantly (*p* < 0.001) increased the percentage of G1 phase cells from 33.85 ± 4.85% (Control) to 51.56 ± 5.69%; however, the incorporation of 0.1 wt.% or 1 wt.% NAC significantly (*p* < 0.001) reduced the percentage of G1 phase cells to 28.97 ± 3.39% and 24.67 ± 5.37%, respectively.

A significant (*p* < 0.05) decrease in S phase cells was observed from 42.98 ± 2.40% (Premier®) to 32.34 ± 3.91% (Control). The incorporation of 0.1 wt.% or 1 wt.% NAC drastically (p < 0.001; p < 0.05) increased the percentage of S-phase cells to 52.32 ± 7.49% and 43.34 ± 4.54%, respectively. The percentages of G2-phase cells were similar in all groups, including controls.

## Discussion

To our knowledge, this study is the first to investigate the cell apoptosis and genotoxicity on HGFs of methacrylate-based dental resin cement Premier® + NAC mixture. Our previous study demonstrated that incorporating NAC (1 wt.%) into dental composite resin may allow a sufficient amount of eluted antioxidant, which consequently reduces the toxicity induced by unpolymerized monomer/co-monomer. Hence, in this study, methacrylate-based resin cement was developed by incorporating the antioxidant NAC as a component, which could also be considered beneficial.

DMEM is an elution medium comparable to saliva which is representative of the oral environmen t[25]. Previous studies investigating cytotoxicity and genotoxicity using only single composite components, such as HEMA and TEGDMA, may have insufficiently investigated the comprehensive effect of eluted components on the surrounding tissue. Thus, in a prior study, we qualified and quantified the eluted components of composite resins that can induce cytotoxicity and DNA-DSBs in HGFs. Therefore, in the present study, the methacrylate-based resin cement with multiple released components in DMEM may reflect the physiological situation.

Our previous study demonstrated that NAC (1 or 0.1 wt.%) incorporated into dental composite resins can be eluted to DMEM, which may exert antiapoptotic and antigenotoxic effects [11, 22]. Additionally, NAC concentrations greater than 50 μM significantly decreased the number of DNA-DSBs generated by dental (co) monomer, intermediates and their epoxy metabolites compared to the corresponding levels in the control [19, 20]. In this study, HGFs were incubated with mixture of methacrylate-based dental resin cement Premier® and NAC. Our results revealed that incorporating NAC diminished cytotoxicity, DNA-DSBs, and cell apoptosis and necrosis; thus, we reasonably speculated that NAC in the Premier® + NAC mixture plays a dominant role on these protective effects. However, according to our results pertaining to ROS detection and γ-H2AX immunofluorescence, the incorporation of NAC (1 wt.%) showed more effective detoxifying effects than NAC (0.1 wt.%). These results indicate that eluted NAC from Premier® + NAC (0.1 wt.%) may not reach effective concentrations.

HGFs were used as suitable model cells because of their direct contact with the eluted monomers immediately following cement-retained implant restoration. HGFs exhibit greater alterations in cell viability following exposure to several luting cements [26, 27]. Exposure of dental resin monomers to HGFs results in cytotoxicity, as demonstrated in a previous study [11]. Multiple mechanisms are involved in cytotoxicity, including oxidative stress, ROS generation, and apoptosis [13]. Furthermore, in vitro and in vivo studies have demonstrated that ROS are strongly associated with bone loss in peri-implantitis [28]. ROS production beyond the cellular redox regulation capacity can result in oxidative stress and cell injury. Chemoattractants, endotoxins, cytokines, and some adhesion molecules play important roles in generating increased oxidative stress [29]; hence, resin cement components released during incomplete polymerization may be considered as a potential factor in triggering peri-implantitis. Incorporating NAC, a ROS scavenger, into methacrylate-based resin cement resulted in an anticytotoxic effect, indicating that the cytotoxicity induced by monomer/comonomers leached from methacrylate-based resin cement is associated with the formation of ROS.

One prior study demonstrated that eluted dental resin components can induce DNA-DSBs in HGFs, and the formation of γ-H2AX foci at the sites of DNA damage is an early step in the cellular response to DNA-DSBs. In the present study, γ-H2AX was detected using immunofluorescence staining. DNA damage is associated with the activation of cell cycle checkpoints [30]. Our data revealed that the use of a resin cement resulted G1-phase cell cycle arrest, in which checkpoints are predominantly activated in response to DNA damage under the present experimental conditions. This is a natural mechanism that protects against further DNA damage by blocking progression through the cell cycle and activating cell cycle checkpoints to allow DNA repair or activation of programmed cell death [31, 32]. In the present study, incorporating the antioxidant NAC resulted in a dose-dependent recovery of resin cement-induced cell cycle arrest, which further indicates that oxidative DNA damage may be the origin of resin cement-induced cell cycle delay. However, in a previous study, HEMA-induced cell cycle arrest was not alleviated by treatment with the antioxidant Trolox [33]; hence, a nonoxidative origin of DNA damage was speculated. These contrasting results may be attributed to the different cell lines selected. However, the precise relationship between oxidative stress and cell cycle arrest currently remains unclear.

The results of the present study indicate that the investigated methacrylate-based dental resin cement Premier® could significantly inhibit the proliferation of HGFs by arresting cells in the G1 phases of the cell cycle, which resulted in ROS production, cell apoptosis, and oxidative DNA damage. Residual methacrylate-based resin cement in the gingival sulcus should raise concerns because of the potential for peri-implant diseases. NAC, a methacrylate-based resin cement component which can be incorporated into methacrylate-based resin cement, diminishes cell cytotoxicity, ROS production, and DNA-DSBs, as well as offers protective effects against the early stage of cell apoptosis and G1 phase arrest of the cell cycle in HGFs.

## Conclusion

Incorporating NAC (1 wt.%) into the methacrylate-based resin cement Premier® diminishes cell cytotoxicity, ROS production, and DNA-DSB production and further offers protective effects against the early stage of cell apoptosis and G1-phase arrest of the cell cycle in HGFs. Overall, our in vitro results indicate that the addition of NAC into methacrylate-based resin cements may have clinically beneficial effects on the cytotoxicity and genotoxicity of these materials.

## Data Availability

All data generated or analyzed during this study are included in this published article.
